# AhR ligands in necrotizing enterocolitis: a provisional evidence map of ligand-specific biology and translational considerations

**DOI:** 10.1080/19490976.2026.2743958

**Published:** 2026-10-23

**Authors:** Laurent Ferraris, Ioana Ferecatu, Julio Aires

**Affiliations:** a Université Paris Cité, Inserm, FPRM, Faculté de Pharmacie de Paris, Paris, France; b FHU Prem’IMPACT Perinatal inflammation and prematurity, Paris, France

**Keywords:** Necrotizing enterocolitis, aryl hydrocarbon receptor, prematurity, microbiota, tryptophan metabolites, indole-3-carbinol, 3-indoleacrylic acid, epithelial necroptosis

## Abstract

Necrotizing enterocolitis (NEC) remains a major cause of gastrointestinal morbidity and mortality in preterm infants, with no targeted mechanism-based therapy. Aryl hydrocarbon receptor (AhR) signaling is an emerging host–microbe interface because it integrates dietary, microbial, milk-derived, and host-derived ligands that regulate intestinal inflammation, epithelial homeostasis, and barrier integrity. Direct NEC-specific evidence for individual AhR ligands, however, remains sparse and uneven. This focused review maps direct NEC intervention evidence, independent replication, human NEC association or ex vivo evidence, AhR dependency, compartment specificity, mechanistic resolution, and translational considerations. Indole-3-carbinol (I3C), the synthetic agonist A18, and 3-indoleacrylic acid (IA) have direct intervention evidence in experimental NEC models. Two independent NEC studies support I3C, although the implicated cellular compartment differs between models. A18 provides direct protection in an experimental NEC model, activates AhR, and exhibits anti-inflammatory effects in human intestinal explants. However, it has not been independently replicated. IA is currently the most comprehensively characterized microbiota-derived ligand in NEC models. However, its mechanistic evidence predominantly comes from one recent study and awaits independent replication. Indole-3-lactic acid, indole-3-acetic acid, and indole-3-sulfate remain physiologically relevant candidates, supported by immature intestinal, human milk, or neonatal metabolic studies. Overall, current evidence supports a ligand-specific view of AhR biology in NEC while highlighting major gaps in replication, human validation, and translational studies.

## Introduction

Necrotizing enterocolitis is one of the most serious gastrointestinal emergencies in neonatology. It primarily affects very preterm and very-low-birth-weight infants and remains associated with substantial mortality and long-term morbidity. Survivors are at increased risk of long-term complications, including intestinal failure and neurodevelopmental impairment.^1^ ^,^ ^2^ Management of established NEC remains largely supportive, and no targeted medical therapy has yet demonstrated clinical benefit.^3-5^ Existing preventive approaches include, human milk feeding, antimicrobial stewardship, and selected probiotic strategies. These approaches may reduce NEC risk in defined settings but do not eliminate disease.^5-7^ Recent experimental data indicate that biofilm-state *Limosilactobacillus reuteri* showed greater intestinal persistence and stronger protection against experimental NEC than planktonic bacteria, and was associated with increased intestinal AhR-ligand production and receptor activation in premature rodents.^8^

Among emerging pathways, aryl hydrocarbon receptor (AhR) signaling has attracted increasing attention. AhR integrates environmental, microbial, dietary, and host-derived ligands and regulates intestinal inflammatory tone, epithelial homeostasis, and barrier responses.^9-11^ Beyond its historical role as a xenobiotic sensor, AhR is also recognized as a physiological integrator of endogenous and environmental signals that can translate ligand exposure into context-dependent cellular responses. This is particularly relevant in the context of NEC because the immature intestine is exposed to rapid changes in colonization, feeding, milk composition, and inflammatory stress. All of these factors may shape the available pool of AhR-active metabolites.^6^ ^,^ ^12^ However, the challenge is that AhR signaling in NEC is often discussed as a single axis. This simplification is no longer adequate. Current data suggest that NEC-relevant ligands differ in source, degree of NEC-specific validation, implicated cellular compartments, and downstream mechanism. Therefore, two principles guide this review. First, the available literature can be mapped according to the type, extent, and depth of evidence supporting each ligand, without assuming that greater mechanistic detail establishes biological superiority. Second, AhR responses appear dependent on ligand-, model-, dose-, developmental context, and cellular compartment rather than being interchangeable.^12-16^

This review is not intended as a general overview of all metabolites linked to NEC. Instead, it addresses a narrower translational question: which specific AhR ligands are directly supported in NEC, through which current cellular targets or tissue compartments do they appear to act, and what evidence gaps and translational considerations should guide future preclinical and early neonatal translational studies?

## Approach to the provisional evidence map

Because the NEC-specific literature is small and heterogeneous, the present framework is intended as a provisional evidence map rather than a quantitative ranking. Several dimensions were considered separately: (1) direct intervention evidence in an experimental NEC model; (2) independent replication across publications or reproducibility across experimental models; (3) human NEC association or *ex vivo* evidence; (4) pharmacological or genetic evidence of AhR dependency; (5) identification of the cellular compartment involved in the observed responses; (6) mechanistic resolution; and (7) translational considerations, including endogenous abundance, stability, delivery feasibility, pharmacokinetics, and safety. These dimensions were not combined into a composite score because mechanistic depth, replication, AhR dependency, and translational feasibility are not equivalent. The resulting map describes where evidence currently exists and where it remains absent (Table 1).

**Table 1.** Provisional evidence map of NEC-relevant AhR ligands.

Ligand or ligand group	Direct NEC intervention evidence	Independent replication	Human NEC association or *ex vivo* evidence	AhR dependency and compartment specificity	Mechanistic resolution	Translational considerations and principal gaps	References
**3-indoleacrylic acid (IA)**	Supplementation protective in mouse and rat NEC models within one publication	Not independently replicated	Reduced fecal IA in infants with NEC; association does not establish causality	Pharmacological AhR inhibition, intestinal epithelial AhR deletion and rescue by AhR reconstitution support epithelial AhR dependency in the reported models	Detailed AhR–SOCS5–STAT1–CYLD pathway associated with reduced RIPK3/MLKL-mediated epithelial necroptosis	Early preclinical candidate; Requires replication, matched comparisons, pharmacokinetics, formulation, dosing, and neonatal safety evaluation	[16]
**Indole-3-carbinol (I3C)**	Direct protection in two experimental NEC studies	Supported by two independent publications, although models, exposure, and outcomes differ	Human premature intestinal tissue evaluated *ex vivo*	Epithelial AhR required in one study but dispensable in another; AhR in CD11c-positive immune cells implicated in the latter model	TLR4-related epithelial injury and cytokine/chemokine-related programs; implicated compartment remains model-dependent	Early preclinical candidate. Proligand generating several acid-condensation products; neonatal product exposure and safety remain undefined	[13,14,17,18]
**Synthetic agonist A18**	Direct protection in one experimental NEC publication	Not independently replicated	Human premature intestinal tissue evaluated *ex vivo*	Pharmacological AhR activation (CYP1A1 induction) and studies in AhR-deficient mice demonstrated; cellular compartment remains incompletely resolved	Suppression of TLR4-associated inflammatory responses demonstrated in NEC-relevant experimental systems	Mechanistic AhR probe; neonatal pharmacokinetics, pregnancy safety, and developmental safety remain undefined	[13]
**3,3′-diindolylmethane (DIM)**	No direct NEC intervention study	Not applicable	No NEC-specific human evidence	AhR activity established outside NEC; no NEC-specific evidence of AhR-dependency or compartment specificity	NEC-specific mechanism unresolved; relevant mainly as a component of the I3C condensation-product mixture	Insufficient NEC-specific evidence; direct intervention, exposure, pharmacokinetic, and safety studies are required	[17,18]
**Indole-3-lactic acid (ILA)**	No direct *in vivo* NEC intervention study; evidence derived from immature enterocytes, NEC-derived cells, and immature intestinal organoids	No independent *in vivo* NEC replication	Immature human intestinal models and associated with early-life microbial metabolism	Pharmacological AhR dependency supported in epithelial experimental systems	Anti-inflammatory activity demonstrated in immature intestinal systems; *in vivo* NEC mechanism and compartment remain unresolved	Exploratory candidate for direct NEC intervention, human milk, microbial production, biomarker, pharmacokinetic, and safety studies	[12,15]
**Indole-3-acetic acid (IAA)**	No direct *in vivo* NEC intervention study	No direct NEC intervention replication	Detected in human milk and evaluated in neonatal intestinal systems	AhR activation and AhR-dependent anti-inflammatory or barrier-supporting effects *in vitro* or *ex vivo*; NEC-specific responding compartment remains unresolved	Functional evidence available in neonatal intestinal systems; NEC-specific mechanistic resolution remains limited	Exploratory candidate for direct NEC intervention, human milk, developmental metabolomics, pharmacokinetic, and safety studies	[12]
**Indole-3-sulfate (I3S)**	No direct *in vivo* NEC intervention study	No NEC intervention replication	Detected in human milk and evaluated in neonatal intestinal systems	AhR activation supported *in vitro*; consistent AhR-dependent protective effects have not been established	Functional effects remain incompletely defined and may differ according to dose, model, and outcome; NEC-specific mechanistic evidence lacking	Exploratory candidate requiring clarification of its inflammatory and intestinal-barrier effects before direct NEC intervention or translational prioritization	[12]

## Indole-3-carbinol and synthetic agonist A18 as first-generation NEC ligands

The first strong evidence that ligand-mediated AhR activation can protect against NEC came from work on indole-3-carbinol (I3C) and the synthetic agonist A18.^13^ In a 2021 study, maternal dietary AhR activation protected offspring from experimental NEC, induced canonical AhR target signaling, and reduced inflammatory outputs in the neonatal intestine.^13^ The same work showed that I3C reduced LPS-induced TNF-alpha expression (a marker of intestinal inflammatory injury) in human intestinal tissue, and identified the synthetic agonist A18 as an AhR agonist that activated receptor signaling and reduced TLR4-associated inflammatory responses (a major driver of epithelial injury and mucosal inflammation) in NEC-relevant systems.^13^ These findings provided direct experimental evidence that AhR activation can modify NEC severity, rather than merely correlating with it. They also introduced an important translational distinction. I3C is a diet-derived proligand with biologic plausibility, whereas the synthetic agonist A18 provides a pharmacological tool for probing receptor activation. This distinction matters for neonatal development because dietary or microbiota-linked ligands and synthetic agonizts may raise different considerations regarding exposure, delivery, pharmacokinetics, and safety, even if they are informative in preclinical models.

The I3C story was refined by later work showing that I3C attenuated experimental NEC, reduced inflammatory mediators including interleukin-1 beta - a key pro-inflammatory cytokine - improved histologic injury, and downregulated cytokine- and chemokine-related pathways.^14^ However, the study did not support a dominant epithelial AhR mechanism in its model.^14^ Mice lacking AhR in intestinal epithelial cells were similarly susceptible to NEC, whereas deletion of AhR in CD11c-positive immune cells increased inflammatory severity and was associated with altered monocyte-macrophage programs.^14^ Thus, I3C is supported by direct intervention evidence from more than one experimental NEC study, but its dominant responding compartment remains unresolved. In the models currently available, one study supports an epithelial AhR-dependent effect, whereas another found that epithelial AhR deletion did not alter NEC susceptibility and implicated AhR signaling in CD11c-positive immune cells.^13^ ^,^ ^14^ These differences may reflect model design, exposure timing, dose, ligand metabolism, or the outcomes examined rather than a fixed ligand-specific cellular target. This distinction has practical implications for study design. In current models, I3C-associated protection should be evaluated using both epithelial and immune readouts rather than being assigned a priori to one compartment. Future studies should determine whether the apparent compartment differences persist when I3C exposure, NEC induction, animal background, and outcome measures are standardized.

## 3,3′-diindolylmethane as an I3C-derived companion ligand

Any NEC review centered on I3C should also address I3C proligand 3,3′-diindolylmethane (DIM). DIM is the major condensation product of I3C under acidic conditions, and both I3C and DIM are recognized AhR ligands.^17^ However, gastric acid condensation produces a mixture rather than a single product. In addition to DIM, I3C can generate other compounds, including the minor high-affinity AhR ligand indolo[3,2-b]carbazole.^17^ ^,^ ^18^ Consequently, effects observed after I3C administration cannot necessarily be assigned to unchanged I3C or to DIM alone. As a result, at least part of the biologic activity attributed to administered I3C may therefore reflect downstream conversion products rather than I3C alone.^17^

DIM has no direct NEC intervention evidence and therefore cannot currently be compared with I3C, A18, or IA on the basis of disease-specific efficacy. Its relevance in this review is primarily interpretive: DIM illustrates how ligand metabolism can complicate attribution of an AhR-dependent response to the administered parent compound.^13-17^

## Host–microbiota tryptophan metabolism as the context for indole ligand availability

The abundance of an individual microbial indole should not be interpreted independently of the broader tryptophan metabolic network. Dietary tryptophan can be metabolized through host-dominated kynurenine and serotonin pathways or through microbiota-dependent pathways that generate IA, ILA, IAA, and other indoles.^19-22^ Inflammation, injury, and infection can alter intestinal indoleamine dioxygenase activity and host tryptophan utilization, while antibiotics, feeding practices, developmental maturity, and microbiota composition can reshape microbial indole production.^20-22^

Longitudinal data from very preterm infants show that feeding type and mode of delivery are associated with differences in fecal host-derived kynurenine metabolites, microbial indoles, and the taxa correlated with these metabolites.^20^ In NEC-sensitive preterm pigs, microbiome suppression by antibiotics shifted systemic tryptophan metabolism toward the kynurenine pathway relative to the serotonin pathway.^21^ These observations do not establish a specific IDO1–kynurenine mechanism in human NEC, but they demonstrate that host and microbial tryptophan pathways are developmentally and environmentally coupled. Reduced concentrations of individual indoles in NEC, such as the reported reduction in IA, could therefore reflect loss of microbial biosynthetic capacity, altered substrate availability, inflammatory redirection of tryptophan metabolism, or a combination of these processes.

Other microbiota-derived indoles with relevant AhR activity include indole, 2-oxindole, and indole-3-propionic acid (IPA). Indole and 2-oxindole are among the dominant fecal AhR-activating tryptophan metabolites, with indole abundant in the gut. Indole is produced by tryptophanase-positive bacteria such as *Escherichia coli*, a common colonizer of the preterm intestine. IPA can also activate AhR, in addition to signaling through pregnane X receptor. However, direct NEC-specific intervention evidence for these metabolites is currently lacking. Their absence from the present evidence map reflects limited NEC-specific data rather than lack of biological relevance. ^23-26^

## 3-indoleacrylic acid as the most comprehensively characterized microbiota-derived ligand

Among microbiota-derived AhR ligands, IA currently has the most detailed NEC mechanistic characterization. A publication reported reduced IA concentrations in fecal samples from infants with NEC and in experimental NEC models. The publication also reported that IA supplementation improved outcomes in mouse and rat NEC models.^16^ These findings provide evidence for direct NEC intervention with IA. However, the level of characterization is not sufficient to establish IA’s biological superiority over other ligands because the results have not yet been independently replicated, and matched head-to-head comparisons have not yet been performed.

Experimental NEC is associated with increased epithelial STAT1 activation and necroptosis, and IA suppresses phosphorylated STAT1, phosphorylated RIPK3, and phosphorylated MLKL in relevant models.^16^ The same study linked this effect to an AhR–SOCS5–STAT1–CYLD pathway, with evidence for direct IA–AhR binding, AhR-dependent induction of SOCS5, increased *Socs5* promoter activity, and a physical SOCS5–STAT1 interaction.^16^ Within the models examined, these experiments support an epithelial AhR–SOCS5–STAT1–CYLD pathway that limits necroptosis.^16^ While this represents the most detailed ligand-specific mechanism reported in NEC, it derives from a single study, and its generalizability across models, species, developmental stages, doses, and exposure conditions remains to be established.^14^ ^,^ ^16^ In the same study, administration of *Bifidobacterium longum* subsp. *infantis* JCM11660, a strain reported to carry an *fldI* homolog, increased intestinal IA concentrations and improved NEC outcomes.^16^ Much of this benefit was lost with AhR inhibition. However, direct IA biosynthesis by *B. infantis* was not demonstrated: the presence of *fldI* alone does not establish the complete phenyllactate dehydratase pathway characterized in validated IA-producing *Peptostreptococcus* and *Clostridium* species.^27^ ^,^ ^28^ *B. infantis* is an established producer of indole-3-lactic acid (ILA).^15^ Thus, the increase in intestinal IA following *B. infantis* administration should be interpreted as an *in vivo* association rather than evidence of strain-level IA production. This finding nevertheless supports further investigation of microbiota-based strategies that modulate intestinal IA availability.. Therefore, IA is best described as the most comprehensively characterized microbiota-derived AhR ligand in current NEC models rather than as a proven superior or clinically prioritized ligand. Its translational relevance remains provisional because independent replication, pharmacokinetics, optimal dosing, formulation, and neonatal safety have not yet been established.

## Indole-3-lactic acid, indole-3-acetic acid, and indole-3-sulfate as physiologic candidates

The importance of IA does not diminish the relevance of other indole ligands. ILA is a physiologically relevant candidate because it has been identified as a *B. longum subsp. infantis*-associated tryptophan metabolite that reduces inflammatory responses in immature enterocytes, NEC-derived enterocytes, and immature organoids. Its AhR dependence has been supported pharmacologically.^15^ These findings support the relevance of ILA to immature gut biology and to the mechanistic link between microbial metabolism and inflammatory susceptibility.^15^

The broader physiologic relevance of indole ligands was further strengthened by a 2024 human milk study showing that ILA, IAA, and indole-3-sulfate (I3S) activate AhR in neonatal gut systems.^12^ AhR-dependent anti-inflammatory and barrier-protective effects were demonstrated most clearly for ILA and IAA. These findings suggest that NEC-relevant AhR biology may not depend on a single dominant ligand, but instead on an early-life metabolite milieu shaped by milk composition, microbial colonization, and developmental maturity.^12^ These studies provide immature-intestinal or neonatal physiological evidence rather than direct *in vivo* NEC intervention evidence. They should therefore be distinguished from ligands tested directly in NEC models. Biofilm-state *L. reuteri* further supports the concept that microbial phenotype and delivery context can influence AhR ligand production and receptor activation during experimental NEC, although the individual active ligands remain incompletely resolved.^8^ Collectively, these studies justify further evaluation of ILA, IAA, and I3S but do not permit conclusions regarding their comparative NEC efficacy.^8^ ^,^ ^12^ ^,^ ^15^

## Provisional evidence map and current compartment-specific interpretations

The current literature supports a provisional evidence map rather than a stable ligand hierarchy. A visual synthesis of these evidence dimensions, current model-supported mechanistic interpretations, and microbial ligand-delivery platforms is provided in Figure 1. I3C, A18, and IA have direct intervention evidence in experimental NEC models, but the type and depth of supporting evidence differ.^13^ ^,^ ^14^ ^,^ ^16^ I3C has been examined in more than one independent NEC study, although the implicated cellular compartment differs between models.^13^ ^,^ ^14^ The synthetic agonist A18 has direct intervention and human *ex vivo* evidence but has not been independently replicated.^13^ IA has the most detailed level of mechanistic characterization and includes human fecal metabolomics associations, two rodent NEC models, pharmacological and genetic AhR validations, and evidence from the epithelial compartment. However, these findings predominantly derive from a single publication.^16^

**Figure 1.**
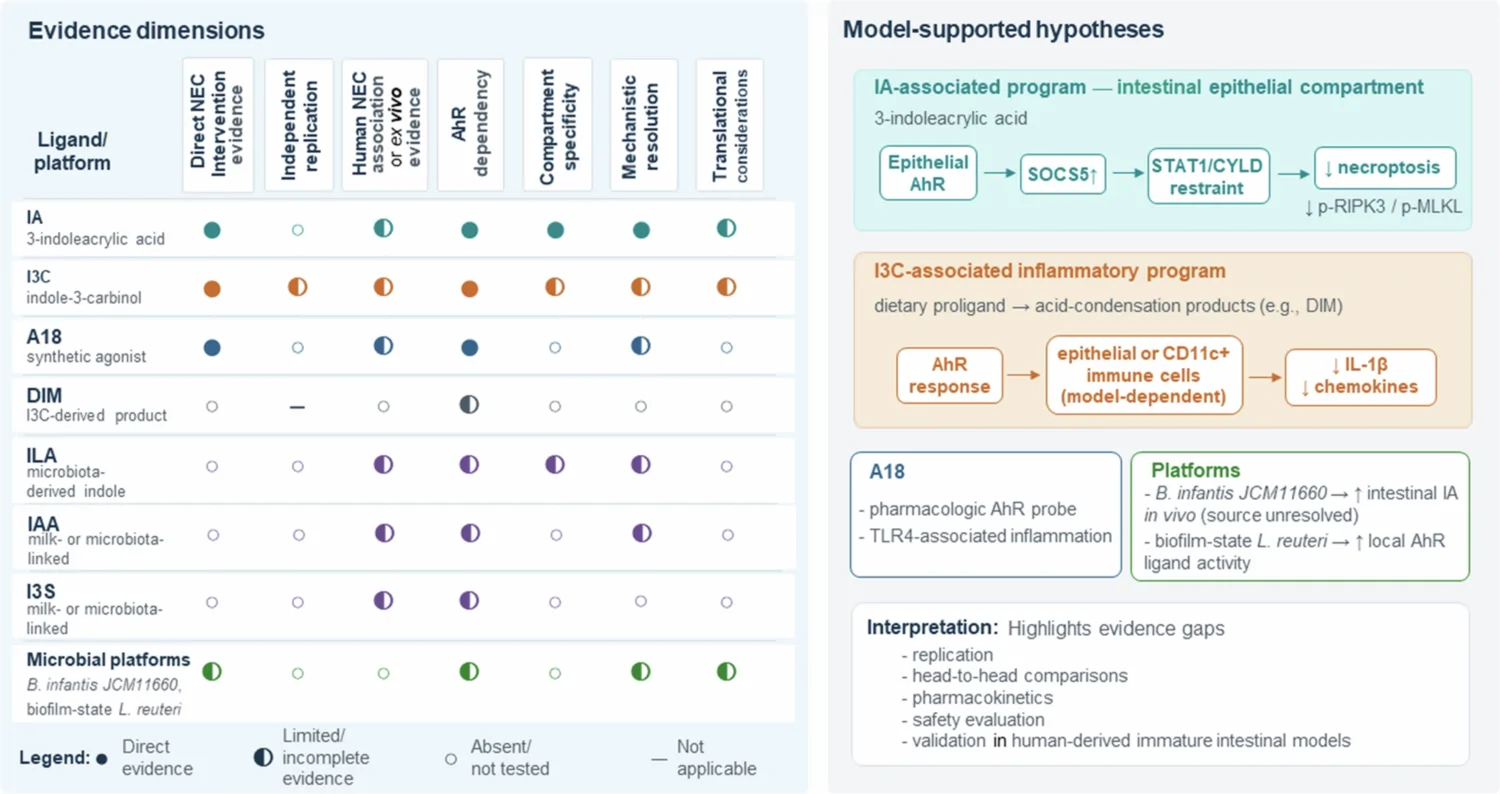
AhR ligands in NEC are presented as a provisional, ligand-specific evidence map—not a definitive ranked hierarchy. Ligands are displayed across distinct evidence dimensions. The symbol matrix provides a visual summary of the evidence dimensions detailed in Table 1; AhR dependency and compartment specificity are displayed separately in the figure for visual clarity. The lower panels illustrate model-supported mechanistic interpretations and microbial ligand-delivery strategies. Filled, partially filled, and open symbols indicate evidence meeting the relevant criterion, limited or incomplete evidence, and absent or untested evidence, respectively; a dash indicates that the category is not applicable. Colors distinguish ligands or strategy groups. The mechanistic pathways shown represent interpretations supported by the models currently available and should not be considered fixed ligand-specific compartment identities. Microbial strategies, including *Bifidobacterium longum subsp. infantis* JCM11660, which was associated with increased intestinal IA *in vivo* without direct demonstration of strain-level IA production, and biofilm-state *Limosilactobacillus reuteri*, are presented as ligand-modulating platforms rather than as individual ligands. IA, 3-indoleacrylic acid; I3C, indole-3-carbinol; DIM, 3,3′-diindolylmethane; ILA, indole-3-lactic acid; IAA, indole-3-acetic acid; I3S, indole-3-sulfate.

DIM has no direct NEC intervention evidence and is included because of its role in I3C metabolism.^17^ ILA, IAA, and I3S are physiologically relevant milk- or microbiota-linked candidates supported by immature intestinal, neonatal, or human milk evidence, but they currently lack direct *in vivo* NEC intervention evidence.^12^ ^,^ ^15^ These categories describe the availability and nature of current data, not ligand potency, biological superiority, or clinical readiness.

Current compartment-specific interpretations should also remain provisional. Available studies suggest immune-cell-associated effects of I3C in one model and an epithelial anti-necroptotic pathway for IA in another.^14^ ^,^ ^16^ These observations generate testable hypotheses but cannot yet establish fixed compartment identities for each ligand. Matched comparisons across ligands, doses, models, and tissue-specific AhR deletions will be required to determine whether these differences are reproducible.

## Translational priorities and research gaps

Biological evidence and translational considerations should be considered separately. IA has the most detailed microbiota-derived mechanistic dataset in current NEC models, but it has not undergone independent replication, neonatal pharmacokinetic testing, formulation development, or safety evaluation.^16^ I3C has evidence from more than one NEC study but is a proligand whose biological effects may reflect variable conversion to multiple products.^13^ ^,^ ^14^ ^,^ ^17^ ^,^ ^18^ The synthetic agonist A18 is useful for receptor probing but lacks dedicated pregnancy, neonatal pharmacokinetic, toxicological, and developmental-safety evaluation.^13^ ILA, IAA, and I3S have physiologic relevance but direct NEC intervention studies are required before their therapeutic potential can be considered.^12^ ^,^ ^15^

Several research gaps now stand out. NEC models should move beyond binary agonist-versus-control designs and directly compare I3C, IA, DIM, and physiologic indoles under matched conditions. Compartment specificity requires more systematic testing, because available data suggest that I3C and IA may act through different cellular compartments.^14^ ^,^ ^16^ Translational work in preterm infants will also require better ligand-linked biomarkers across stool, milk, serum, urine, and intestinal tissue compartments.^6^ ^,^ ^12^

A further important distinction is that the NEC-specific provisional evidence map should not be equated with intrinsic AhR agonist potency. AhR ligands vary widely in apparent affinity and functional potency, from high-affinity ligands active in the pico- to nanomolar range to weaker or indirect activators requiring micromolar concentrations.^19^ ^,^ ^29^ This is particularly relevant for I3C, which can generate multiple acid-condensation products, including DIM and the indolo[3,2-b]carbazole (ICZ), despite ICZ being present as a minor condensation product.^17-19^ By contrast, the synthetic agonist A18 was functionally identified as a pharmacological AhR activator in NEC models, with dose-dependent AhR-reporter activation and CYP1A1 induction at micromolar concentrations, but available NEC data do not define a binding affinity or allow direct potency ranking against IA, I3C-derived metabolites, or physiologic indoles.^13^ Therefore, future NEC studies should distinguish ligand potency, stability, local concentration, metabolic conversion, tissue exposure, and compartment-specific biological response rather than assuming that AhR-active compounds are interchangeable.

Ligand identity may influence AhR output independently of apparent agonist potency. Structurally distinct ligands can induce selective transcriptional responses and differ in metabolic stability, exposure duration, receptor activity, and canonical versus noncanonical signaling outputs.^19^ ^,^ ^29^ ^,^ ^30^ Species-dependent AhR ligand selectivity cautions against direct quantitative extrapolation from murine models to human neonatal systems.^31^ Translational evaluation should therefore include human-derived intestinal models and compare different ligands, concentrations, exposure durations, target-gene profiles, and functional outcomes rather than relying on CYP1A1 induction alone.

Biomarker strategies should be compartment-specific. Stool measurements may estimate luminal metabolite availability and microbial biosynthetic capacity but may not reflect epithelial exposure or systemic absorption. Human milk measurements characterize an external ligand source, whereas plasma and urine integrate absorbed microbial metabolites and host kynurenine and serotonin metabolism. Intestinal CYP1A1 expression can provide a proximal pharmacodynamic marker of AhR activation but is unlikely to be routinely accessible in preterm infants. Longitudinal studies should therefore combine stool metabolomics and microbial functional genes with paired milk, plasma, or urine measurements and clinical variables. Results from one compartment should not be assumed to represent AhR activity in another.^12^ ^,^ ^20^ ^,^ ^32^

Before neonatal application, all IA- and I3C-based strategies will require rigorous pharmacokinetic, dose-response, formulation, toxicology, and developmental-safety studies.^6^ ^,^ ^16^ Translation from rodent NEC models is further limited by differences in intestinal maturation, microbial assembly, experimental disease induction, and human versus mouse AhR pharmacology.^31^ Among potential translational approaches, carefully defined probiotic or postbiotic strategies that modify local luminal ligand production could be investigated as an alternative to systemic administration of potent purified agonizts. Even these approaches should initially be viewed as hypothesis-generating and should include long-term monitoring of growth, immune development, infection risk, and neurodevelopment. The biofilm-state *L. reuteri* study supports proof of concept that probiotic formulation can alter intestinal persistence, AhR ligand production, and receptor activation during experimental NEC.^8^

## Conclusions

The NEC-specific AhR literature remains early, heterogeneous, and unevenly distributed among ligands. The principal contribution of this review is therefore a provisional evidence map rather than a definitive hierarchy. I3C, A18, and IA have direct NEC intervention evidence, but differ in replication, human NEC association or *ex vivo* evidence, mechanistic resolution, and translational considerations. IA is currently the most comprehensively characterized microbiota-derived ligand in NEC models, but this characterization derives predominantly from one study and does not establish superiority. I3C has evidence from multiple NEC studies, although its cellular mechanism appears model-dependent. DIM remains relevant to interpretation of I3C exposure but lacks direct NEC evidence, whereas ILA, IAA, and I3S remain physiologically plausible candidates requiring further disease-specific validation.

The evidence map should evolve as independent replication, matched ligand comparisons, human intestinal studies, pharmacokinetic data, and neonatal safety evidence become available. Priority experiments include head-to-head ligand testing under standardized conditions, systematic tissue-specific AhR deletion studies, integrated host–microbial tryptophan metabolomics, and validation in human-derived immature intestinal models. The key question is not simply which ligand has the most detailed current dataset, but which ligand, dose, exposure route, and cellular context provide reproducible benefit with acceptable developmental safety.

## Data Availability

Does not apply.
